# Dissociable effects of psilocybin and escitalopram for depression on processing of musical surprises

**DOI:** 10.1038/s41380-025-03035-8

**Published:** 2025-04-26

**Authors:** Rebecca Harding, Neomi Singer, Matthew B. Wall, Talma Hendler, David Erritzoe, David Nutt, Robin Carhart-Harris, Leor Roseman

**Affiliations:** 1 Centre for Psychedelic Research, Division of Brain Sciences, Imperial College London, London, UK; 2 Clinical Psychopharmacology Unit, University College London, London, UK; 3 Sagol Brain Institute and the Department of Neurology, Tel Aviv Sourasky Medical Center, Tel Aviv, Israel; 4 Perceptive, Centre for Imaging Sciences, London, UK; 5 Departments of Neurology & Psychiatry, University of California San Francisco, San Francisco, CA USA; 6 Department of Psychology, University of Exeter, Exeter, UK

**Keywords:** Neuroscience, Psychology

## Abstract

Psilocybin therapy (PT) is emerging as an effective intervention for Major Depressive Disorder (MDD), offering comparable efficacy to conventional treatments like selective serotonin reuptake inhibitors (SSRIs). Music, an emotionally evocative stimulus, provides a valuable tool to explore changes in hedonic and predictive processing mechanisms via expectancy violations, or ‘surprises’. This study sought to compare behavioural and functional magnetic resonance imaging (fMRI) responses to musical surprises in MDD patients treated with either PT or the SSRI, escitalopram. In this secondary analysis of a trial, 41 MDD patients (with usable fMRI data) were randomly assigned to either PT (*n* = 22) or escitalopram (*n* = 19) treatment groups. Participants listened to music during fMRI and tracked their emotional experience, both before and after a 6-week intervention. Surprise-related valence and arousal indices were calculated. Musical surprises were entered as regressors for whole-brain and region of interest fMRI analyses. PT caused a greater decrease in anhedonia scores compared with escitalopram. While escitalopram led to reductions in surprise-related affective responses, PT showed no significant change. Escitalopram was associated with increased activation in memory and emotional processing areas during musical surprises (versus control events) when compared with PT. Following PT, there was decreased activation in the ventromedial prefrontal cortex and angular gyrus, and greater activation in sensory regions. PT may allow for the subjective response to musical surprises to be maintained through a lasting reduction in the salience of prediction errors, or, alternatively, by increasing hedonic priors. Contrastingly, escitalopram may diminish hedonic priors, highlighting fundamental differences in treatment mechanisms.

## Introduction

Major Depressive Disorder (MDD) is a common mood disorder that is characterised by pathological emotional and hedonic processing [1]. Anhedonia, defined as a loss of pleasure in response to ordinarily hedonic stimuli, is a core feature of MDD that occurs due to dysfunction in reward processing circuitry [2, 3], particularly the mesocorticolimbic pathway [4–6]. This circuit connects regions in the ventral striatum, including the nucleus accumbens (NAc), to higher cortical areas including the ventromedial prefrontal cortex (vmPFC), an area involved in the higher-order cognitive processing of emotion and integration of salience signals about reward [7]. Alterations in functioning within these regions may therefore illuminate the neural underpinnings of anhedonia and indicate potential treatment mechanisms.

Selective serotonin reuptake inhibitors (SSRIs) are currently the recommended first-line treatment for MDD. Escitalopram is a particularly selective serotonin reuptake inhibitor with a relatively good tolerability to efficacy profile [8]. However, despite SSRIs being widely prescribed, they demonstrate limited efficacy, and around 50% of patients report “emotional blunting” side effects; a restricted range or intensity of emotional experiences [9]. In addition, SSRIs often fall short in addressing the symptom of anhedonia, which is a particularly difficult symptom to treat [10, 11]. Conversely, psilocybin therapy (PT) is a promising intervention for the treatment of MDD [12, 13], which has been shown to reduce symptoms of anhedonia post-acutely [14] and mediate persisting changes in brain activation and connectivity in response to emotional stimuli [15, 16]. Moreover, a significant treatment difference was found between escitalopram and PT on experiential avoidance, indicating the putative dissociable treatment effects on processing emotional and rewarding stimuli [12].

Psilocybin (4-phosphoryloxy-N, N-dimethyltryptamine) is the prodrug of psilocin, a classic ‘psychedelic’ compound that initiates its principal subjective effects via agonism of 5-HT_2A_ receptors. These receptors are found abundantly on deep pyramidal cells in the cortex [17]. In line with the Bayesian predictive coding framework [18], deep pyramidal cell functioning has been linked with the encoding of top-down predictive processing [19], i.e. encoding implicit assumptions. As described by the Relaxed Beliefs Under pSychedelics (REBUS) model [20], acute dysregulation of neuronal assemblages associates directly with a decrease in the weighting or influence of top-down predictive processes encoding assumptions or ‘beliefs’. In the context of depression, this may allow individuals with over-weighted maladaptive beliefs a degree of respite during which they might begin to revise and de-weight these assumptions [21]. Yet, despite the acute phase of psilocybin lasting 3–6 h [22], the clinical benefits of PT are often sustained for many months [14, 23, 24]. It is speculated that the enduring de-weighting of over-weighted or canalized assumptions may underlie the putative enduring efficacy of PT [21].

Music is a powerful, emotionally evocative, often hedonic stimulus [25]. The experience of music-induced pleasure has been associated with the recruitment of nodes in the mesocorticolimbic pathway that are associated with hedonic processing [26–28]. One phenomenon that purportedly mediates the emotional experience of music is expectancy [29–35]. This may be described as the establishment of predictions regarding future auditory events, and their subsequent fulfilment or violation [36, 37]. Expectancy violations, referred to here as musical surprises, are associated with pleasantness felt during music listening [38, 39], offering an excellent naturalistic paradigm to explore changes in hedonic processing. Additionally, the Geneva Emotional Music Scale (GEMS), previously used in our lab to demonstrate changes in processing of emotion and pleasure following PT [40], offers a broader framework for assessing music-evoked emotional responses. The GEMS categorises these responses into three dimensions: sublimity, vitality, and unease [41]. *Sublimity* captures transcendent emotions such as awe and wonder, *vitality* represents positively valenced, high-arousal states associated with excitement and energy, and *unease* encompasses low-valence emotions related to tension or discomfort [42]. Of particular interest, vitality may contrast with the low-valence, low-arousal state characteristic of anhedonia [43]. By combining an analysis of responses to musical surprises with music-evoked emotions, we adopt a holistic and naturalistic framework for examining treatment effects on hedonic and emotional processes, spanning from short-term hedonic changes to shifts in overall affective tone.

From a predictive coding viewpoint, musical surprises provide new information that may refine an individual’s prediction of future events (i.e. prediction error) and inform how rewarding they are [44]. This reward prediction error is purportedly encoded by mesocorticolimbic regions that are similarly triggered upon anticipation or consumption of reward [29, 32]. Specifically, increased activation has been seen in the NAc during the experience of musical pleasure [38, 45, 46], and recently, greater subjective liking of the music has been further linked to the response of this region to musical surprise. For example, the so-called ‘chills’ response to music has been linked with musical surprise [47], and musical surprises have been associated with positive changes in emotion-induced responses [39]. Additionally, higher cortical areas including the superior temporal gyrus (STG), an area involved in the sensory processing of music, and the vmPFC, which is associated with the higher-order processing of emotion, have been implicated in the processing of musical surprises and music-evoked pleasure and emotion [38, 39, 48, 49]. Therefore, given their distinct yet complementary roles, investigating surprise-related changes in these regions may offer insight into the varying treatment mechanisms of escitalopram and PT on symptoms of anhedonia and predictive processing. Previous work has also shown that changes in responses to musical stimuli can be sensitive and informative markers of PT [40, 50].

The overall aim of this study was to examine if treatment with escitalopram or PT in patients with MDD affected subjective hedonic and neurobehavioural responses to musical surprises. We predicted that PT would show higher efficacy at improving symptoms of anhedonia compared with escitalopram. Further, we predicted that escitalopram would cause a significant decrease in surprise-related pleasantness, whereas PT would cause an increase. Specifically, an increase in the activation of the NAc during surprising events was hypothesised for the PT condition. Additionally, we predicted significantly different surprise-related activation in the vmPFC, auditory sensory (STG), and mesolimbic (NAc) areas between escitalopram and PT, due to differences in treatment effects on reward and emotional processing.

## Materials and methods

### Study design

This was a specific fMRI analysis carried out on data from a phase II, double-blind, randomised, controlled trial involving the allocation of participants with MDD to either PT or escitalopram treatment groups; the main study report has been previously published [12]. A total of 59 patients were enrolled but 50 were included in this present analysis, with nine lost due to an incomplete dataset owing to COVID-19 lockdowns. 26 participants were randomised to the PT condition and 24 to the escitalopram condition. Of these participants, five were removed as a result of excessive movement in the fMRI scanner or deviation from the protocol. Overall, a total of 19 patients in the escitalopram group and 22 in the PT group were available for analysis. Further details of participant criteria and trial procedures can be found in Carhart Harris et al. [12].

MRI scanning was performed before any therapeutic intervention (pre-treatment, i.e., one-day prior to the first dosing session) and six weeks and one day after the first dosing day (three weeks after the second psilocybin dosing session). Protocols on scanning days were identical, with completion of the fMRI scan, followed by the continuous music rating task and the self-reported subjective measures of music-evoked emotion and anhedonia (see below).

### fMRI music task and image acquisition

The fMRI task involved music stimuli featuring a piano arrangement of “The Hours” by Phillip Glass (423 secs long). Rest/baseline blocks were also included, with 1 min of silence before and after the song; the total task time was 510 s. Music was played through magnetic-resonance compatible headphones which were built in-house. Participants were instructed to lay still inside the scanner with their eyes closed while listening to the music. Participants were asked to complete a continuous music rating task immediately after the scan (see Supplementary Methods).

fMRI data were acquired using a Siemens TIM Trio 3 Tesla MRI scanner (Siemens, Erlangen), equipped with a 32-channel phased-array head coil. Anatomical images were acquired using the recommended parameters for MPRAGE by the ADNI-GO project: TE = 2.98 ms, TR = 2300 ms, 160 sagittal slices, 256 × 256 in-plane FOV, flip angle = 9°, 1 mm isotropic voxels. The functional (T2*-weighted gradient echo Echo-Planar-Imaging) acquisition was based on the multiband EPI WIP v012b provided by the University of Minnesota using a multiband acceleration factor of 2, and a slice acceleration (GRAPPA) factor of 2 (TR = 1250 ms, TE = 30 ms, 44 slices, 3 mm isotropic voxels, FOV = 192 × 192 mm, flip angle = 70°, bandwidth = 2232 Hz/pixel, 408 volumes acquired). This was based on sequences previously tested and validated by [51].

### Subjective measures

#### Musical-emotion label ratings

Immediately following the continuous music ratings (see Supplementary Methods), participants completed the 25-item Geneva Emotional Music Scale (GEMS-25) [41] to determine the general affective tone that was evoked in listeners. This questionnaire includes items that reflect musically induced emotional states rated from 1–5 on a Likert Scale. Items are averaged into nine emotion subscales which are further be organised into three factors: (1) Sublimity: wonder, transcendence, power, tenderness, nostalgia, peacefulness, (2) Vitality: joyful activation, (3) Unease: sadness and tension.

#### Anhedonia

Following the music task, participants completed the 15-item Snaith-Hamilton Pleasure Scale (SHAPS) [52] to assess the extent of anhedonia using a Likert scale from 0–3, where 0 = strongly disagree and 3 = strongly agree.

### Analysis

#### BOLD fMRI analysis

The preprocessing pipeline was the same as previously used by Carhart-Harris et al. [53] and based on Shany et al. [39] (See Supplementary Methods). For the first-level analyses, a standard general linear model (GLM) was used to model the effect of surprising events on whole-brain voxel-wise BOLD activation, as implemented in the FEAT module in FSL. Surprising events in the song were annotated and ranked by professional musicians in a separate study [39], where additional details of the recording, processing, and annotation of the music and surprising events can be found. The most highly surprising events (based on a median split of annotated events) and unsurprising events (randomly selected) were used as regressors (see Supplementary Material for exact details of event times). Three regressors of interest were entered into subject level GLMs, consisting of two experimental conditions: (1) surprising events (test condition; n_events_ = 17), (2) unsurprising events (control condition; n_events_ = 17), and (3) the remaining music. These stimulus-related time series were convolved with the canonical Gamma function to model the haemodynamic response. Modelling used the FILM module in FSL for pre-whitening and autocorrelation correction. Contrasts were computed that compared surprising with unsurprising events.

Mid-level fixed-effects individual-subject analyses were used to generate comparisons between pre- and post-treatment scans for each subject. Between-subjects group-level mixed-effects analyses were performed using FSL’s FLAME (FSL’s Local Analysis of Mixed Effects) 1 + 2) on the mid-level beta values to compare the treatment groups using unpaired t-test contrasts of PT vs escitalopram groups. These group-level analyses therefore represent a 2 (pre vs. post therapy; contrasted at the mid-level) by 2 (treatment group; contrasted at the group level) interaction analysis. Additional group-level analyses were conducted for each treatment group separately, and these used the first-level analyses as inputs to conduct within-group paired t-tests (pre>post and post>pre). All group-level statistical maps were thresholded at Z = 2.3 and *p* < 0.05 (cluster corrected). Group level images were visualised on an average MNI 0.5 mm surface brain using MRIcron [54].

##### ROI analyses

Three a priori ROIs were chosen based on structures that have been commonly identified for music-evoked pleasure (NAc and vmPFC) and auditory perception (STG) [35, 39]. Based on previous literature that indicates the right NAc is a more dependable indicator of musically evoked emotion [55], a 6 mm spherical ROI was derived based on the coordinates provided by Shany et al. [39], (MNI coordinates 11,9,−1). Moreover, the 10 mm spherical vmPFC seed was taken from a study by Mas-Herrero et al. [56] (MNI coordinates −2,46,−8). Lastly, a region encompassing the right STG was created by multiplying a binarized version of the mean task effect (see Supplementary Methods) by the right STG assigned by the Harvard-Oxford probabilistic atlas (provided with FSL) to ensure task-specificity.

#### Continuous music ratings

Continuous ratings of valence and arousal (collected after the scan) were extracted per subject and down-sampled into a resolution of 1 Hz. This was performed using MATLAB (R2021a, MathWorks).

##### Event-related affective change

To assess how emotional experience was transiently modulated by musical surprise, changes in arousal and valence were measured on a second-by-second basis. These changes were quantified as positive (increase) or negative (decrease) values of the first derivative of the corresponding rating (i.e. change per second), as per the protocol used by Shany et al. [39]. This resulted in four time series per subject, depicting valence-increase, valence-decrease, arousal-increase, and arousal-decrease, which was further standardised into Z-scores within each subject. Following this, an event-related affective change index was calculated by separately taking the mean of each of the four valence and arousal changes occurring during 1–4 s following the onset of all surprising or unsurprising events, as previous research has indicated this is likely an optimal time-frame for capturing such responses [33, 39, 57]. The mean event-related affective change indices per subject were extracted and a two-tailed paired t-test was performed to assess whether there was an event-related change (surprising vs. unsurprising events) in each of the four indices to validate the paradigm. The significance level (alpha) was adjusted to 0.01 to account for multiple comparisons.

## Results

### Anhedonia and subjective music ratings

**Fig. 1:**
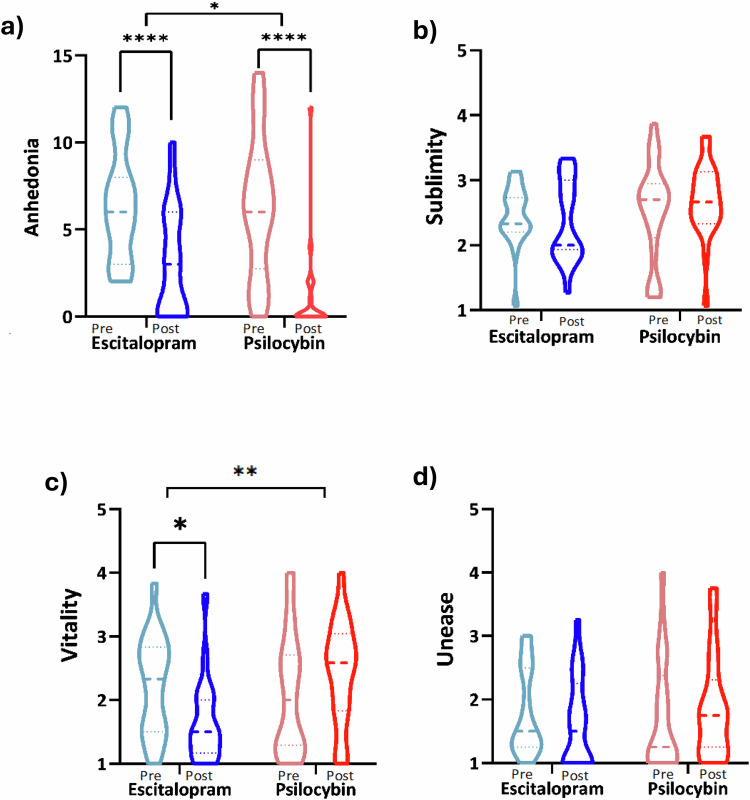
Subjective measures of anhedonia and music ratings. **a** Anhedonia (SHAPS) scores at pre-treatment and post-treatment for escitalopram (*n* = 19) and PT (*n* = 22). Emotional responses to music (GEMS) scores for **b** sublimity **c** vitality and **d** unease. Results are shown with median and quartile ranges depicted. Results of interaction test post-hoc analyses are shown. ****p ≤ 0.0001; **p ≤ 0.001 *p ≤ 0.05. Escitalopram = blue, PT =  red. Pre-treatment = light colour, post-treatment = dark colour.

A decrease in anhedonia (SHAPS) scores was seen in both escitalopram (mean = −3.211, SEM = 0.5952) and PT (mean = −5.273; SEM = 0.7845) (Fig. 1b). A mixed-effects model showed a significant interaction between *treatment* and *time* (*p* = 0.0480; F(1, 39) = 4.170) on anhedonia scores. *Post hoc* analyses showed a significant decrease in anhedonia scores in both escitalopram (t(18) = 5.394, *p* < 0.0001) and PT (t(21) = 6.721; *p* < 0.0001) from pre-treatment to post-treatment, however the interaction result implies that PT had a significantly larger effect (Fig. 1a).

Furthermore, a mixed-effects model revealed a significant interaction between *treatment* and *time* in music-evoked vitality scores (F(1,39) = 7.967, *p* = 0.0075) (Fig. 1b). *Post hoc* analyses showed a significant decrease (mean = −0.5702, SEM = 0.2292) in vitality from pre-treatment to post-treatment in escitalopram (t(18) = 2.488, *p* = 0.0229), although the observed increase in vitality in PT (mean=0.333; SEM = 0.222) was not significant (t(21) = 1.50, *p* = 0.1482), implying that the decrease under escitalopram that was the more likely cause of the between-condition difference. There was no significant effect of *treatment* or *time* on subjective ratings of sublimity (Fig. 1b) and unease (Fig. 1d) felt during music listening (*p* > 0.05).

### Changes in subjective experience associated with musical surprises

**Fig. 2:**
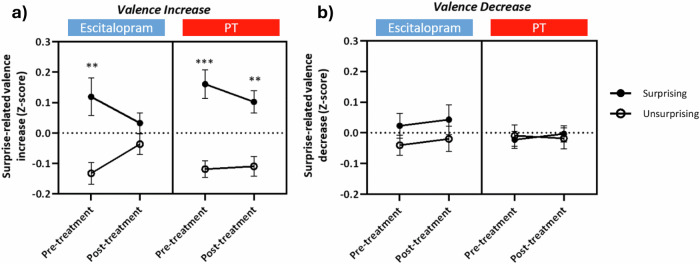
Changes in subjective experience associated with musical surprises. Event-related affective change index for surprising and unsurprising events for escitalopram (*n* = 19) and PT (*n* = 22) at pre-treatment and post-treatment for **a** Valence Increase **b** Valence Decrease. Shown as mean ± SEM for surprising (*n* = 17) and unsurprising (*n* = 17) events. Asterisks represent paired t-test results comparing Z-scores for surprising to unsurprising events **p ≤ 0.01 ***p ≤ 0.001.

Event-related affective change indices were calculated from each time-course of valence and arousal (mean time-courses for each treatment group can be seen in Supplementary Fig. S2). To establish validity and replicate a previous study [39], we looked at differences in surprise-related change between surprising and unsurprising events in each group, with the significance level adjusted to 0.01 to account for multiple comparisons. Results of a two-tailed paired t-test demonstrate that surprising events caused a significant transient increase in valence compared to unsurprising events at pre-treatment in both escitalopram (t(18) = 3.011; *p* = 0.0075) and PT (t(21) = 4.134; *p* = 0.0005) groups (Fig. 2a), thereby validating the paradigm. Surprise-related increases in valence remain robust post-PT, with surprising events showing significantly greater valence than unsurprising ones (t(21) = 3.818, *p* = 0.0010). In contrast, the escitalopram condition exhibits marked changes post-treatment: valence rises for unsurprising events while declining for surprising ones. This shift abolishes the previously significant difference between event types (*p* = 0.2202), suggesting a modulation of emotional responses that diminishes the distinction between surprising and unsurprising stimuli. Moreover, there was no significant surprise-related valence decrease at pre-treatment or post-treatment in either escitalopram or PT (*p* > 0.05; Fig. 2b). Results for arousal can be found in Supplementary Fig. S3.

### BOLD fMRI

#### ROI analyses

**Fig. 3:**
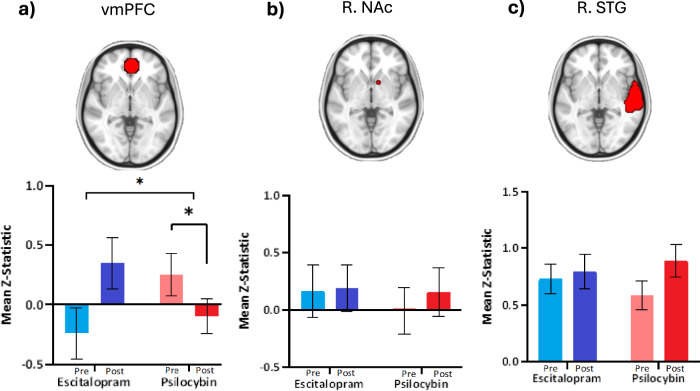
ROI analyses at pre-treatment (blue) and post-treatment (red) for escitalopram (*n* = 19) and PT (*n* = 22). Mean ± SEM BOLD activation (Z-statistics) for surprising events compared to unsurprising events (surprise>unsurprise) shown for (**a**) vmPFC (**b**) R. NAc and (**c**) R. STG (seen in red on brain image). Results of interaction and post-hoc simple effects analysis are depicted. *p ≤ 0.05. Escitalopram arm = blue, PT arm = red. Baseline = light colour, endpoint = dark colour.

A significant interaction between *treatment* and *time* (F(1,39) = 7.074, *p* = 0.0113) on surprise-related activation (surprise>unsurprise) was observed in the vmPFC (Fig. 3a). A *post-hoc* simple effects analysis demonstrated no significant effect in escitalopram from pre- to post-treatment (t(18) = −1.767, *p* = 0.0941), despite a numerical increase in vmPFC activation post-escitalopram, while a significant decrease in surprise-related activation of the vmPFC was observed post-PT (t(21) = 2.195, *p* = 0.0395). Importantly, the results of a two-tailed unpaired t-test revealed no significant difference in baseline (pre-treatment) vmPFC activation between escitalopram and PT (t(39) = 1.791, *p* = 0.0810), however, a marginal trend suggesting differences in baseline was observed. No significant interaction was observed between *treatment* and *time* (*p* = 0.7655), nor a significant effect of *treatment* (*p* = 0.7655) or *time* (*p* = 0.6746), on activation of the right NAc in response to surprising versus unsurprising events (Fig. 3b). Finally, there was no significant interaction (*p* = 0.3575), nor effect of *treatment* (*p* = 0.872) or *time* (*p* = 0.163), in surprise-related activation of the STG (Fig. 3c). No significant correlations between surprise-related BOLD in these ROIs and subjective measures (anhedonia & music ratings) or valence increase were observed.

#### Whole-brain analyses

**Fig. 4:**
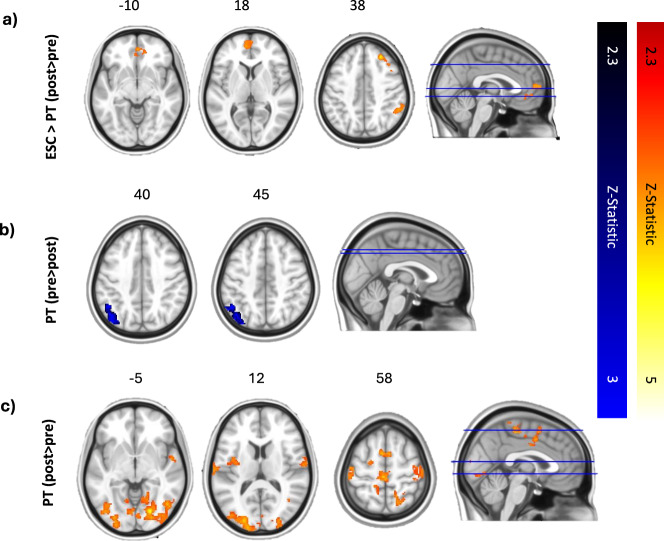
Brain regions showing altered BOLD response to surprising>unsurprising events in ESC (escitalopram; *n* = 19) and PT (*n* = 22) between pre and post-treatment. **a** Results of an interaction analysis comparing across the two time-points (pre vs. post therapy) and the two treatment groups (PT vs. escitalopram), depicting areas where there is an observed interaction effect (i.e. significant increase in activation post-therapy in the escitalopram group compared to the PT, where there is instead a significant decrease following therapy). **b** Results of a paired-samples t-test (within-group; pre > post-treatment) performed on results for treatment effect of PT, showing a relative decrease post-treatment in activity after therapy in the left angular gyrus. **c** Results of a paired-samples t-test (within-group; post > pre-treatment) performed on results for treatment effect of PT showing relative increases post-treatment in sensory (visual, auditory) cortex and a number of other regions – see text for details. All analyses were thresholded at Z > 2.3, *p* < 0.05, cluster-corrected for multiple comparisons. Shown in MNI space and neurological orientation, i.e. left of the image = left hemisphere.

The mean treatment group activation for surprising versus unsurprising events can be seen before and after treatment in escitalopram and PT in Supplementary Fig. S4. As expected, cortical regions associated with music listening and auditory processing were activated during the task in all groups, including the superior temporal gyrus, Heschl’s gyrus, and the planum temporale. Results of a two-sample unpaired t-test on first-level analyses (within-subject differences from pre- to post-treatment) showed significantly increased activation in escitalopram and decreased activation post PT in the right angular gyrus, right posterior supramarginal gyrus, frontal pole, middle gyrus, frontal medial cortex, paracingulate gyrus (escitalopram>PT; Fig. 4a). No significant regions were identified where increased activation was observed in PT compared to escitalopram (PT>escitalopram).

Results of a paired t-test showed no significant within-group treatment effect in the escitalopram group on BOLD response to surprising compared with unsurprising events. In the PT group, significant widespread increases in BOLD were observed post-treatment in the bilateral lateral occipital cortex, bilateral occipital fusiform gyrus, occipital pole, and right postcentral gyrus, right precentral gyrus, central opercular cortex, and bilateral clusters in the superior temporal gyrus (Fig. 4c). Post-treatment reductions in activity were also observed following PT in the left lateral occipital cortex extending to the angular gyrus (Fig. 4b).

## Discussion

This study sought to evaluate the treatment differences between escitalopram and PT on the affective and neural correlates of musical surprises in patients with MDD. Notably, both treatments were shown to be significantly effective at treating symptoms of anhedonia, with PT demonstrating more robust efficacy. Escitalopram reduced music-evoked feelings of vitality, while PT enhanced them. Surprise-related subjective hedonic response was decreased in the escitalopram condition, whereas there was no change with PT, implying either a blunting effect of escitalopram on hedonic responsiveness or a (relative) enhancement with PT. Meanwhile, regarding brain responses, significantly less surprise-related vmPFC activation was seen with PT versus escitalopram, as seen using a between-condition interaction test. Finally, in a within-condition analysis, increases in activations to surprising musical events in regions associated with visual and sensory processing and decreases in angular gyrus activity were seen post-PT.

Contrary to our a priori hypothesis, there was no significant change in surprise-related activation of the NAc in either treatment group. This was unexpected given that the music literature ascribes an intrinsic role to the NAc in surprise-related pleasantness and prediction error coding [39, 44]. One possible explanation may be that there is reduced NAc activation to musical stimuli in depression [58]. Additionally, this observed lack of effect may be owing to the idiosyncratic nature of music-evoked reward, whereby music taste is influenced by factors including personality traits, intelligence, and musical training [59, 60]. Future studies may attempt to acknowledge the intrinsic variation in music-evoked reward by stratifying treatment groups at baseline by high and low pleasantness ratings [39].

In this study, the PT group showed a significant decrease in the surprise-related activation of the vmPFC relative to the escitalopram condition. Also, in the PT condition only, increased post-treatment activations were seen in sensory regions. From a predictive coding standpoint, the precision weighting or salience of prediction errors, or surprises, is thought to occur in the higher cortical regions such as the vmPFC, where the higher cortex is mandated to process bottom-up information flow [7]. Consequently, a reduction in activation in high level cortex, as seen here in the vmPFC, may reflect a lack of confidence (precision weighting) regarding the incoming prediction error signal. Alterations in salience processing may occur as a lasting consequence of the rapid and dramatic reduction in the precision weighting of top-down predictions hypothesised to occur in the acute psychedelic state [20]. Indeed, 5-HT2A receptors are heavily expressed on deep pyramidal cells in the vmPFC [61, 62] where 5-HT_2A_R agonism would dysregulate ensemble activity there [63] and in parallel, decrease precision weighting of top-down predictive processes. It is possible that the post-acute decrease in vmPFC responses to musical surprise observed in the PT condition of this study reflect a lasting decrease in the precision weighting of top-down priors. We acknowledge that this interpretation is speculative, however.

Additionally, it has been suggested that increased vmPFC activity in MDD may portray a pathological increase in the executive control of emotion [64]. This may indicate a reduction in the overly enhanced control over affective responses, which may serve as a potential therapeutic mechanism of PT wherein a reduction in top-down control allows for an increase in the bandwidth of emotions experienced [15, 16]. This interpretation is supported by a recent finding from a previous study in treatment-resistant depression that following PT, there is a reduction of music-induced coupling of the NAc to areas in the default mode network (DMN), including the vmPFC [40]. The authors suggested this may enable a recovery of a normal hedonic response, consistent with other findings of increased brain responses to emotional stimuli post-PT [16]. Another interpretation is that a decrease in the responsiveness of the vmPFC to surprise, along with the increase in sensory processing, may reflect a switch from excessively internal processing [65] to a more external, sensory driven mode of processing that may be characteristic of recovery from depression [66].

It is important to note that while there is a significant overlap, there is a distinct difference between perceptual processing, in which future musical events are predicted, and reward processing, which predicts the rewards for future events [67]. Sensory processing is linked to musical aesthetics, whereas reward processing relies on reinforcement learning to maximise reward value that is irrespective of structural specifics [68]. Alterations in the STG functioning was explored due to its role in monitoring auditory possibilities [32, 38] and these results demonstrate no treatment difference in the surprise-related activation of the STG and incidental sensory processing of musical surprises. However, reward is a complex psychophysiological construct and the exact interaction between sensory and reward processing of music remains to be adequately studied.

Aside from the a priori ROIs which were selected based on their involvement in the processing of musical surprises [39], results from exploratory whole-brain analyses showed that PT caused an increase in the surprise-related activation of areas that serve visual functions, e.g. the bilateral occipital cortex [69], and sensory integration functions e.g. the central opercular cortex [70]. Interestingly, symptoms of depression and anhedonia are associated with a decreased ability to discriminate between different sensory stimuli [71, 72]. This increased sensory activation may therefore reveal a potential target of PT with regards to hedonic processing. Furthermore, the post-PT reduction in activation of the angular gyrus is intriguing, particularly owing to its role in the default mode network and as a cross-modal hub where converging multisensory information is combined and integrated [73]. This decrease in activity could suggest a reduction in the cognitive integration of sensory information, allowing for a stronger focus on raw sensory processing during musical surprises. In line with this, our between-group analyses revealed that, compared to escitalopram, the PT condition was associated with decreased activation in areas including the posterior supramarginal gyrus, frontal pole, paracingulate gyrus, and again the angular gyrus. These areas play a role in mediating attention, memory during music listening, and personal familiarity with music [74–76]. Given that attention is considered to be the cognitive homologue of adding precision weighting or salience to prediction errors [77], this aligns with our current findings that PT causes a shift in the salience of prediction errors, e.g., de-weighting salience processed via the vmPFC and other high level cortical regions.

While it is intriguing to consider the role of predictive processing, our behavioural findings related to changes in valence towards surprising events may suggest an alternative hypothesis: PT might enhance the reward response to hedonic stimuli. Instead of reducing priors, PT could increase hedonic priors and positive expectations for surprising events, potentially explaining the sustained positive valence observed. Although clinical research on this is limited, pre-clinical animal models have shown that psilocybin can increase optimism bias, with enhanced belief updating after positive outcomes, such as greater reward responses following wins [78]. Consistent with this alternative hypothesis, our data suggest that escitalopram may heighten valence responses to previously neutral stimuli (i.e. unsurprising events), and potentially diminish reward responses to surprising events in the absence of a hedonic prior. This aligns with previous research that escitalopram reduces learning from reward and enhances learning from punishment [79]. However, this interpretation remains speculative and warrants further investigation.

To our knowledge, this is the first study to explore the processing of musical surprises in MDD specifically and may offer key insight into alterations in reward processing that can occur post-treatment. Compared with previous studies that have used no music as a control, one of the strengths of this study is that it used other parts of the music as a naturalistic comparator. However, this study had a relatively small sample size and participants listened to only one song inside the scanner, thus the replicability of these findings may be limited. This study would have additionally benefited from objective physiological readings for arousal to further validate these self-reported behavioural measures. Validation via other hedonic stimuli than just music would also have added confidence, if they replicated the main findings presented here with musical surprise.

Moreover, participants were required to listen to the same musical piece on multiple occasions throughout the study, thus potentially impacting the response to musical surprises via a familiarity effect. Furthermore, surprising events were annotated separately by professional musicians and subjective level of surprise was not reported by the study group themselves. Additionally, as anticipated, the musical piece used in this study predominantly elicited positive valence in response to surprising elements, which was selected due to its relevance in reward processing in depression. However, musical surprises are more broadly linked to changes in subjective experience [39] and can also evoke negative valence [33, 80–82]. To better understand how these interventions influence predictive coding mechanisms, future research should incorporate a broader range of stimuli that includes those capable of inducing negative valence. This approach is especially pertinent given evidence suggesting that PT has lasting effects on affective and neural responses to negative stimuli. [83]. Indeed, given the important and influential role that music plays in PT [84], future analyses may wish to consider persisting differences in music perception based on an individual’s subjective music experience during the acute drug state.

PT has been observed to have profound antidepressant effects, with a potentially superior efficacy to SSRIs for MDD, particularly in alleviating anhedonia. [85, 86]. In line with this, we found neurobiological correlates of the hedonic processing of music differed in patients treated with PT e.g. with less activation in the vmPFC activation and increased activation in sensory regions. A follow-up analysis of directed connectivity might test the hypothesis that there is greater bottom-up information flow from the sensory regions post treatment with PT. This has been observed during the acute LSD state, with increased parahippocampal cortex–visual cortex functional connectivity and information flow during music listening [87]. Persisting alteration in functional connectivity has been previously observed, with decreased amygdala-occipital/parietal cortices connectivity during face processing post-PT [15]. Indeed, the present findings could be regarded as suggestive evidence that PT decreases the top-down executive control of emotions in an enduring way, as has been reported qualitatively and quantitatively with PT for depression [88]. The reduction by escitalopram of the affective response to musical surprises, fits with fMRI measured brain responses to other emotional stimuli [89], thus supporting intrinsic differences in the therapeutic mechanism of SSRIs versus those of PT.

## Supplementary information

Supplementary material

## Data Availability

Data may be available upon request by contacting Leor Roseman at l.roseman@exeter.ac.uk.

## References

[1] Pizzagalli DA. Depression, stress, and anhedonia: toward a synthesis and integrated model. Annu Rev Clin Psychol. 2014;10:393–423. 24471371 10.1146/annurev-clinpsy-050212-185606 PMC3972338

[2] Borsini A, Wallis ASJ, Zunszain P, Pariante CM, Kempton MJ. Characterizing anhedonia: a systematic review of neuroimaging across the subtypes of reward processing deficits in depression. Cogn Affect Behav Neurosci. 2020;20:816–41. 32472419 10.3758/s13415-020-00804-6 PMC7395022

[3] Whitton AE, Treadway MT, Pizzagalli DA. Reward processing dysfunction in major depression, bipolar disorder and schizophrenia. Curr Opin Psychiatry. 2015;28:7–12. 25415499 10.1097/YCO.0000000000000122 PMC4277233

[4] Argyropoulos SV, Nutt DJ. Anhedonia revisited: is there a role for dopamine-targeting drugs for depression? J Psychopharmacol Oxf Engl. 2013;27:869–77. 10.1177/0269881113494104 23904408

[5] Coccurello R. Anhedonia in depression symptomatology: appetite dysregulation and defective brain reward processing. Behav Brain Res. 2019;372:112041. 31220485 10.1016/j.bbr.2019.112041

[6] Stanton CH, Holmes AJ, Chang SWC, Joormann J. From stress to anhedonia: molecular processes through functional circuits. Trends Neurosci. 2019;42:23–42. 30327143 10.1016/j.tins.2018.09.008 PMC6344037

[7] Bartra O, McGuire JT, Kable JW. The valuation system: a coordinate-based meta-analysis of BOLD fMRI experiments examining neural correlates of subjective value. NeuroImage. 2013;76:412–27. 23507394 10.1016/j.neuroimage.2013.02.063 PMC3756836

[8] Cleare A, Pariante CM, Young AH, Anderson IM, Christmas D, Cowen PJ, et al. Evidence-based guidelines for treating depressive disorders with antidepressants: A revision of the 2008 British Association for Psychopharmacology guidelines. J Psychopharmacol Oxf Engl. 2015;29:459–525. 10.1177/0269881115581093 25969470

[9] Goodwin GM, Price J, De Bodinat C, Laredo J. Emotional blunting with antidepressant treatments: a survey among depressed patients. J Affect Disord. 2017;221:31–5. 28628765 10.1016/j.jad.2017.05.048

[10] Corruble E, de Bodinat C, Belaïdi C, Goodwin GM. Efficacy of agomelatine and escitalopram on depression, subjective sleep and emotional experiences in patients with major depressive disorder: a 24-wk randomized, controlled, double-blind trial. Int J Neuropsychopharmacol. 2013;16:2219–34. 23823799 10.1017/S1461145713000679

[11] Farabaugh A, Fisher L, Nyer M, Holt D, Cohen M, Baer L, et al. Similar changes in cognitions following cognitive-behavioral therapy or escitalopram for major depressive disorder: Implications for mechanisms of change. Ann Clin Psychiatry Off J Am Acad Clin Psychiatr. 2015;27:118–26. 25954938

[12] Carhart-Harris R, Giribaldi B, Watts R, Baker-Jones M, Murphy-Beiner A, Murphy R, et al. Trial of psilocybin versus escitalopram for depression. N Engl J Med. 2021;384:1402–11. 33852780 10.1056/NEJMoa2032994

[13] Davis AK, Barrett FS, May DG, Cosimano MP, Sepeda ND, Johnson MW, et al. Effects of psilocybin-assisted therapy on major depressive disorder. JAMA Psychiatry. 2021;78:1–9. 33146667 10.1001/jamapsychiatry.2020.3285 PMC7643046

[14] Carhart-Harris RL, Bolstridge M, Rucker J, Day CMJ, Erritzoe D, Kaelen M, et al. Psilocybin with psychological support for treatment-resistant depression: an open-label feasibility study. Lancet Psychiatry. 2016;3:619–27. 27210031 10.1016/S2215-0366(16)30065-7

[15] Mertens LJ, Wall MB, Roseman L, Demetriou L, Nutt DJ, Carhart-Harris RL. Therapeutic mechanisms of psilocybin: changes in amygdala and prefrontal functional connectivity during emotional processing after psilocybin for treatment-resistant depression. J Psychopharmacol. 2020;34:167–80. 31941394 10.1177/0269881119895520

[16] Roseman L, Demetriou L, Wall MB, Nutt DJ, Carhart-Harris RL. Increased amygdala responses to emotional faces after psilocybin for treatment-resistant depression. Neuropharmacology. 2018;142:263–9. 29288686 10.1016/j.neuropharm.2017.12.041

[17] Beliveau V, Ganz M, Feng L, Ozenne B, Højgaard L, Fisher PM, et al. A high-resolution in vivo atlas of the human brain’s serotonin system. J Neurosci. 2017;37:120–8. 28053035 10.1523/JNEUROSCI.2830-16.2016 PMC5214625

[18] Friston K. The free-energy principle: a rough guide to the brain? Trends Cogn Sci. 2009;13:293–301. 19559644 10.1016/j.tics.2009.04.005

[19] Friston K. Hierarchical models in the brain. PLOS Comput Biol. 2008;4:e1000211. 18989391 10.1371/journal.pcbi.1000211 PMC2570625

[20] Carhart-Harris RL, Friston KJ. REBUS and the anarchic brain: toward a unified model of the brain action of psychedelics. Pharmacol Rev. 2019;71:316–44. 31221820 10.1124/pr.118.017160 PMC6588209

[21] Carhart-Harris RL, Chandaria S, Erritzoe DE, Gazzaley A, Girn M, Kettner H, et al. Canalization and plasticity in psychopathology. Neuropharmacology. 2023;226:109398. 36584883 10.1016/j.neuropharm.2022.109398

[22] Passie T, Seifert J, Schneider U, Emrich HM. The pharmacology of psilocybin. Addict Biol. 2002;7:357–64. 14578010 10.1080/1355621021000005937

[23] Carhart-Harris RL, Bolstridge M, Day CMJ, Rucker J, Watts R, Erritzoe DE, et al. Psilocybin with psychological support for treatment-resistant depression: six-month follow-up. Psychopharmacology. 2018;235:399–408. 29119217 10.1007/s00213-017-4771-x PMC5813086

[24] Griffiths RR, Johnson MW, Carducci MA, Umbricht A, Richards WA, Richards BD, et al. Psilocybin produces substantial and sustained decreases in depression and anxiety in patients with life-threatening cancer: a randomized double-blind trial. J Psychopharmacol Oxf Engl. 2016;30:1181–97. 10.1177/0269881116675513 PMC5367557 27909165

[25] Juslin PN, Laukka P. Expression, perception, and induction of musical emotions: A review and a Questionnaire study of Everyday Listening. J New Music Res. 2004;33:217–38.

[26] Blood AJ, Zatorre RJ. Intensely pleasurable responses to music correlate with activity in brain regions implicated in reward and emotion. Proc Natl Acad Sci. 2001;98:11818–23. 11573015 10.1073/pnas.191355898 PMC58814

[27] Koelsch S, Fritz T, v. Cramon DY, Müller K, Friederici AD. Investigating emotion with music: An fMRI study. Hum Brain Mapp. 2006;27:239–50. 16078183 10.1002/hbm.20180 PMC6871371

[28] Zatorre RJ. Musical pleasure and reward: mechanisms and dysfunction. Ann N Y Acad Sci. 2015;1337:202–11. 25773636 10.1111/nyas.12677

[29] Gebauer L, Kringelbach ML, Vuust P. Ever-changing cycles of musical pleasure: The role of dopamine and anticipation. Psychomusicology Music Mind Brain. 2012;22:152–67.

[30] Huron D, Margulis EH. Musical expectancy and thrills. In P. N. Juslin & J. A. Sloboda (Eds.), Handbook of music and emotion: Theory, research, applications. New York, NY, US: Oxford University Press; 2010. p. 575–604.

[31] Meyer LB. Emotion and meaning in music. Chicago: University of Chicago Press; 1956.

[32] Salimpoor VN, Zald DH, Zatorre RJ, Dagher A, McIntosh AR. Predictions and the brain: how musical sounds become rewarding. Trends Cogn Sci. 2015;19:86–91. 25534332 10.1016/j.tics.2014.12.001

[33] Steinbeis N, Koelsch S, Sloboda JA. The role of harmonic expectancy violations in musical emotions: evidence from subjective, physiological, and neural responses. J Cogn Neurosci. 2006;18:1380–93. 16859422 10.1162/jocn.2006.18.8.1380

[34] Vuust P, Heggli OA, Friston KJ, Kringelbach ML. Music in the brain. Nat Rev Neurosci. 2022;23:287–305. 35352057 10.1038/s41583-022-00578-5

[35] Zatorre RJ, Salimpoor VN. From perception to pleasure: music and its neural substrates. Proc Natl Acad Sci. 2013;110:10430–7. 23754373 10.1073/pnas.1301228110 PMC3690607

[36] Huron D. Sweet anticipation: music and the psychology of expectation. Cambridge, MA: MIT Press; 2008. p. 477.

[37] Siman-Tov T, Granot RY, Shany O, Singer N, Hendler T, Gordon CR. Is there a prediction network? Meta-analytic evidence for a cortical-subcortical network likely subserving prediction. Neurosci Biobehav Rev. 2019;105:262–75. 31437478 10.1016/j.neubiorev.2019.08.012

[38] Salimpoor VN, van den Bosch I, Kovacevic N, McIntosh AR, Dagher A, Zatorre RJ. Interactions between the nucleus accumbens and auditory cortices predict music reward value. Science. 2013;340:216–9. 23580531 10.1126/science.1231059

[39] Shany O, Singer N, Gold BP, Jacoby N, Tarrasch R, Hendler T, et al. Surprise-related activation in the nucleus accumbens interacts with music-induced pleasantness. Soc Cogn Affect Neurosci. 2019;14:459–70. 30892654 10.1093/scan/nsz019 PMC6523415

[40] Shukuroglou M, Roseman L, Wall M, Nutt D, Kaelen M, Carhart-Harris R. Changes in music-evoked emotion and ventral striatal functional connectivity after psilocybin therapy for depression. J Psychopharmacol. 2023;37:70–9. 36433778 10.1177/02698811221125354 PMC9834320

[41] Zentner M, Grandjean D, Scherer KR. Emotions evoked by the sound of music: characterization, classification, and measurement. Emotion. 2008;8:494–521. 18729581 10.1037/1528-3542.8.4.494

[42] Trost W, Ethofer T, Zentner M, Vuilleumier P. Mapping aesthetic musical emotions in the brain. Cereb Cortex. 2012;22:2769–83. 22178712 10.1093/cercor/bhr353 PMC3491764

[43] Kerns JG, Docherty AR, Martin EA. Social and physical anhedonia and valence and arousal aspects of emotional experience. J Abnorm Psychol. 2008;117:735–46. 19025222 10.1037/a0013601

[44] Gold BP, Pearce MT, Mas-Herrero E, Dagher A, Zatorre RJ. Predictability and uncertainty in the pleasure of music: a reward for learning? J Neurosci. 2019;39:9397–409. 31636112 10.1523/JNEUROSCI.0428-19.2019 PMC6867811

[45] Blood AJ, Zatorre RJ, Bermudez P, Evans AC. Emotional responses to pleasant and unpleasant music correlate with activity in paralimbic brain regions. Nat Neurosci. 1999;2:382–7. 10204547 10.1038/7299

[46] Salimpoor VN, Benovoy M, Larcher K, Dagher A, Zatorre RJ. Anatomically distinct dopamine release during anticipation and experience of peak emotion to music. Nat Neurosci. 2011;14:257–62. 21217764 10.1038/nn.2726

[47] Grewe O, Nagel F, Kopiez R, Altenmüller E. Listening to music as a re-creative process: physiological, psychological, and psychoacoustical correlates of chills and strong emotions. Music Percept Interdiscip J. 2007;24:297–314.

[48] Kim SG, Mueller K, Lepsien J, Mildner T, Fritz TH. Brain networks underlying aesthetic appreciation as modulated by interaction of the spectral and temporal organisations of music. Sci Rep. 2019;9:19446. 31857651 10.1038/s41598-019-55781-9 PMC6923468

[49] Koelsch S. Towards a neural basis of music-evoked emotions. Trends Cogn Sci. 2010;14:131–7. 20153242 10.1016/j.tics.2010.01.002

[50] Wall MB, Lam C, Ertl N, Kaelen M, Roseman L, Nutt DJ, et al. Increased low-frequency brain responses to music after psilocybin therapy for depression. J Affect Disord. 2023;333:321–30. 37094657 10.1016/j.jad.2023.04.081

[51] Demetriou L, Kowalczyk OS, Tyson G, Bello T, Newbould RD, Wall MB. A comprehensive evaluation of increasing temporal resolution with multiband-accelerated protocols and effects on statistical outcome measures in fMRI. NeuroImage. 2018;176:404–16. 29738911 10.1016/j.neuroimage.2018.05.011

[52] Snaith RP, Hamilton M, Morley S, Humayan A, Hargreaves D, Trigwell P. A scale for the assessment of hedonic tone the snaith–hamilton pleasure scale. Br J Psychiatry. 1995;167:99–103. 7551619 10.1192/bjp.167.1.99

[53] Carhart-Harris RL, Muthukumaraswamy S, Roseman L, Kaelen M, Droog W, Murphy K, et al. Neural correlates of the LSD experience revealed by multimodal neuroimaging. Proc Natl Acad Sci USA. 2016;113:4853–8. 27071089 10.1073/pnas.1518377113 PMC4855588

[54] Rorden C, Brett M. Stereotaxic display of brain lesions. Behav Neurol. 2000;12:191–200. 11568431 10.1155/2000/421719

[55] Koelsch S. Brain correlates of music-evoked emotions. Nat Rev Neurosci. 2014;15:170–80. 24552785 10.1038/nrn3666

[56] Mas-Herrero E, Dagher A, Farrés-Franch M, Zatorre RJ. Unraveling the temporal dynamics of reward signals in music-induced pleasure with TMS. J Neurosci. 2021;41:3889–99. 33782048 10.1523/JNEUROSCI.0727-20.2020 PMC8084325

[57] Sloboda JA, Lehmann AC. Tracking performance correlates of changes in perceived intensity of emotion during different interpretations of a chopin piano prelude. Music Percept. 2001;19:87–120.

[58] Jenkins LM, Skerrett KA, DelDonno SR, Patrón VG, Meyers KK, Peltier S, et al. Individuals with more severe depression fail to sustain nucleus accumbens activity to preferred music over time. Psychiatry Res Neuroimaging. 2018;275:21–7. 29555382 10.1016/j.pscychresns.2018.03.002 PMC5899937

[59] Chamorro-Premuzic T, Swami V, Cermakova B. Individual differences in music consumption are predicted by uses of music and age rather than emotional intelligence, neuroticism, extraversion or openness. Psychol Music. 2012;40:285–300.

[60] Rentfrow PJ, Gosling SD. The do re mi’s of everyday life: the structure and personality correlates of music preferences. J Pers Soc Psychol. 2003;84:1236–56. 12793587 10.1037/0022-3514.84.6.1236

[61] Madsen MK, Fisher PM, Burmester D, Dyssegaard A, Stenbæk DS, Kristiansen S, et al. Psychedelic effects of psilocybin correlate with serotonin 2A receptor occupancy and plasma psilocin levels. Neuropsychopharmacology. 2019;44:1328–34. 30685771 10.1038/s41386-019-0324-9 PMC6785028

[62] Vollenweider FX, Vontobel P, Hell D, Leenders KL. 5-HT modulation of dopamine release in basal ganglia in psilocybin-induced psychosis in man—A PET Study with [11C]raclopride. Neuropsychopharmacology. 1999;20:424–33. 10192823 10.1016/S0893-133X(98)00108-0

[63] Celada P, Puig MV, Díaz-Mataix L, Artigas F. The hallucinogen DOI reduces low-frequency oscillations in rat prefrontal cortex: reversal by antipsychotic drugs. Biol Psychiatry. 2008;64:392–400. 18436196 10.1016/j.biopsych.2008.03.013

[64] Koenigs M, Grafman J. The functional neuroanatomy of depression: distinct roles for ventromedial and dorsolateral prefrontal cortex. Behav Brain Res. 2009;201:239–43. 19428640 10.1016/j.bbr.2009.03.004 PMC2680780

[65] Zhou HX, Chen X, Shen YQ, Li L, Chen NX, Zhu ZC, et al. Rumination and the default mode network: meta-analysis of brain imaging studies and implications for depression. NeuroImage. 2020;206:116287. 31655111 10.1016/j.neuroimage.2019.116287

[66] Cash RFH, Müller VI, Fitzgerald PB, Eickhoff SB, Zalesky A. Altered brain activity in unipolar depression unveiled using connectomics. Nat Ment Health. 2023;1:174–85.

[67] Hansen NC, Dietz MJ, Vuust P. Commentary: predictions and the brain: how musical sounds become rewarding. Front Hum Neurosci. 2017;11:168. 28424603 10.3389/fnhum.2017.00168 PMC5380745

[68] Schultz W. Updating dopamine reward signals. Curr Opin Neurobiol. 2013;23:229–38. 23267662 10.1016/j.conb.2012.11.012 PMC3866681

[69] Malach R, Reppas JB, Benson RR, Kwong KK, Jiang H, Kennedy WA, et al. Object-related activity revealed by functional magnetic resonance imaging in human occipital cortex. Proc Natl Acad Sci. 1995;92:8135–9. 7667258 10.1073/pnas.92.18.8135 PMC41110

[70] Tanaka S, Kirino E. The parietal opercular auditory-sensorimotor network in musicians: a resting-state fMRI study. Brain Cogn. 2018;120:43–7. 29122368 10.1016/j.bandc.2017.11.001

[71] Atanasova B, El-Hage W, Chabanet C, Gaillard P, Belzung C, Camus V. Olfactory anhedonia and negative olfactory alliesthesia in depressed patients. Psychiatry Res. 2010;176:190–6. 20207422 10.1016/j.psychres.2008.11.016

[72] Colle R, El Asmar K, Verstuyft C, Lledo PM, Lazarini F, Chappell K, et al. The olfactory deficits of depressed patients are restored after remission with venlafaxine treatment. Psychol Med. 2020;1–9. 10.1017/S0033291720003918. 10.1017/S0033291720003918 33087184

[73] Seghier ML. The angular gyrus: multiple functions and multiple subdivisions. The Neuroscientist. 2013;19:43–61. 22547530 10.1177/1073858412440596 PMC4107834

[74] Bashwiner DM, Wertz CJ, Flores RA, Jung RE. Musical creativity “Revealed” in brain structure: interplay between motor, default mode and limbic networks. Sci Rep. 2016;6:20482. 26888383 10.1038/srep20482 PMC4757893

[75] Castro M, L’héritier F, Plailly J, Saive AL, Corneyllie A, Tillmann B, et al. Personal familiarity of music and its cerebral effect on subsequent speech processing. Sci Rep. 2020;10:14854. 32908227 10.1038/s41598-020-71855-5 PMC7481778

[76] Tanaka S, Kirino E. Increased functional connectivity of the angular gyrus during imagined music performance. Front Hum Neurosci. 2019;13:92. 30936827 10.3389/fnhum.2019.00092 PMC6431621

[77] Hohwy J. The predictive mind. Oxford, UK: OUP Oxford; 2013. p. 293.

[78] Fisher EL, Smith R, Conn K, Corcoran AW, Milton LK, Hohwy J, et al. Psilocybin increases optimistic engagement over time: computational modelling of behaviour in rats. Transl Psychiatry. 2024;14:1–10. 39349428 10.1038/s41398-024-03103-7 PMC11442808

[79] Michely J, Eldar E, Erdman A, Martin IM, Dolan RJ. Serotonin modulates asymmetric learning from reward and punishment in healthy human volunteers. Commun Biol. 2022;5:1–9. 35962142 10.1038/s42003-022-03690-5 PMC9374781

[80] Koelsch S, Kilches S, Steinbeis N, Schelinski S. Effects of unexpected chords and of performer’s expression on brain responses and electrodermal activity. PloS ONE. 2008;3:e2631. 18612459 10.1371/journal.pone.0002631 PMC2435625

[81] Koelsch S, Fritz T, Schlaug G. Amygdala activity can be modulated by unexpected chord functions during music listening. Neuroreport. 2008;19:1815–9. 19050462 10.1097/WNR.0b013e32831a8722

[82] Egermann H, Pearce MT, Wiggins GA, McAdams S. Probabilistic models of expectation violation predict psychophysiological emotional responses to live concert music. Cogn Affect Behav Neurosci. 2013;13:533–53. 23605956 10.3758/s13415-013-0161-y

[83] Barrett FS, Doss MK, Sepeda ND, Pekar JJ, Griffiths RR. Emotions and brain function are altered up to one month after a single high dose of psilocybin. Sci Rep. 2020;10:2214. 32042038 10.1038/s41598-020-59282-y PMC7010702

[84] Kaelen M, Giribaldi B, Raine J, Evans L, Timmerman C, Rodriguez N, et al. The hidden therapist: evidence for a central role of music inpsychedelic therapy. Psychopharmacology. 2018;235:505–19. 29396616 10.1007/s00213-017-4820-5 PMC5893695

[85] Barba T, Buehler S, Kettner H, Radu C, Cunha BG, Nutt DJ, et al. Effects of psilocybin versus escitalopram on rumination and thought suppression in depression. BJPsych Open. 2022;8:e163. 36065128 10.1192/bjo.2022.565 PMC9534928

[86] Weiss B, Erritzoe D, Giribaldi B, Nutt DJ, Carhart-Harris RL. A critical evaluation of QIDS-SR-16 using data from a trial of psilocybin therapy versus escitalopram treatment for depression. J Psychopharmacol. 2023;37:717–32. 37122239 10.1177/02698811231167848 PMC10350722

[87] Kaelen M, Roseman L, Kahan J, Santos-Ribeiro A, Orban C, Lorenz R, et al. LSD modulates music-induced imagery via changes in parahippocampal connectivity. Eur Neuropsychopharmacol. 2016;26:1099–109. 27084302 10.1016/j.euroneuro.2016.03.018

[88] Watts R, Kettner H, Geerts D, Gandy S, Kartner L, Mertens L, et al. The Watts Connectedness Scale: a new scale for measuring a sense of connectedness to self, others, and world. Psychopharmacology. 2022;239:3461–83. 35939083 10.1007/s00213-022-06187-5 PMC9358368

[89] Wall MB, Demetriou L, Giribaldi B, Roseman L, Ertl N, Erritzoe D, et al. Reduced brain responsiveness to emotional stimuli with escitalopram but not psilocybin therapy for depression. medRxiv. 2023 [cited 2023 Aug 23]. Available from: https://www.medrxiv.org/content/10.1101/2023.05.29.23290667v1. 10.1176/appi.ajp.20230751 40329640

