# Correction: Dissociable effects of psilocybin and escitalopram for depression on processing of musical surprises

**DOI:** 10.1038/s41380-025-03066-1

**Published:** 2025-06-06

**Authors:** Rebecca Harding, Neomi Singer, Matthew B. Wall, Talma Hendler, David Erritzoe, David Nutt, Robin Carhart-Harris, Leor Roseman

**Affiliations:** 1 Centre for Psychedelic Research, Division of Brain Sciences, Imperial College London, London, UK; 2 Clinical Psychopharmacology Unit, University College London, London, UK; 3 Sagol Brain Institute and the Department of Neurology, Tel Aviv Sourasky Medical Center, Tel Aviv, Israel; 4 Perceptive, Centre for Imaging Sciences, London, UK; 5 Departments of Neurology & Psychiatry, University of California San Francisco, San Francisco, CA USA; 6 Department of Psychology, University of Exeter, Exeter, UK

**Keywords:** Neuroscience, Psychology

Correction to: *Molecular Psychiatry* 10.1038/s41380-025-03035-8, published online 26 April 2025

The following part of the abstract had a minor error: ‘Following PT, there was greater activation in the ventromedial prefrontal cortex and sensory regions, and reduced activation in the angular gyrus.’ This is now corrected to ‘Following PT, there was decreased activation in the ventromedial prefrontal cortex and angular gyrus, and greater activation in sensory regions.’ The original article has been updated accordingly.

