# Supplementary material for: Dissociable effects of psilocybin and escitalopram for depression on processing of musical surprises
Source: Mol Psychiatry. 2025 Apr 26;30(7):3188–96. doi: 10.1038/s41380-025-03035-8 (PMC12185350; doi:10.1038/s41380-025-03035-8)
Supplement: Supplementary file 1 — Supplementary material [file 41380_2025_3035_MOESM1_ESM.docx]

# Supplemental information

**Contents**

**Supplementary data**

***Figures***

**Figure S1.** Timeline of experimental events

**Figure S2.** Average timecourses for valence and arousal

**Figure S3.** Association of musical surprises with changes in arousal

**Figure S4.** Mean activation for each treatment group before and after intervention

***Tables***

**Table S1.** **Details of experimental events**.

**Table S2.** Characteristics of each treatment group

**Supplemental Methods**

**Supplemental References**

## Supplementary data

**Figure S1 Timeline of experimental events**


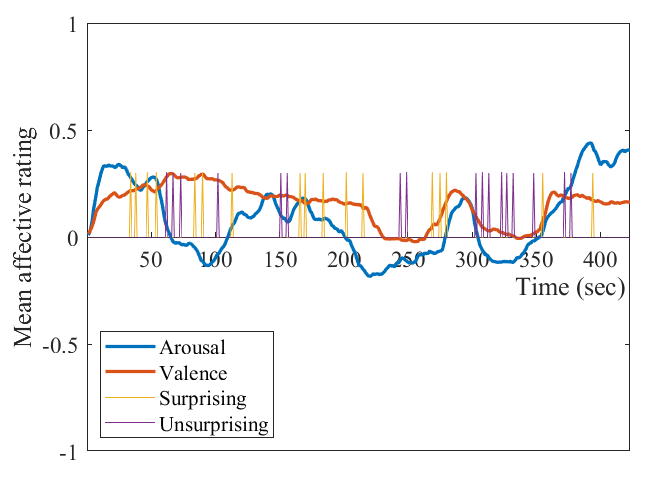


Figure S1 shows the distribution of surprising and unsurprising events during the music musical piece. Occurrence of surprising and unsurprising events is marked by spikes, with each event lasting 1 second (yellow for surprising events, purple for unsurprising events – the height and width are arbitrary). The grand mean of the continuous ratings of valence (red) and arousal (blue) are overlaid on the plot of each piece as well. The Y axis depicts the mean of each of these affective ratings, and the X axis depicts time in seconds (sec). The exact timings of the surprising and unsurprising events are found in table 1.

**Table 1. Details of experimental events**. In each row of the table, the exact event onset in seconds is given, ranked from most highly surprising according to annotators at the top of the table. Unsurprising events were not ranked.

| **Surprising event onset (s) (n=17)** | **Unsurprising event onset (s) (n=17)** |
| --- | --- |
| 275.952 (most highly surprising) | 61.887 |
| 166.032 | 67.499 |
| 215.605 | 72.908 |
| 54.535 | 102.322 |
| 84.004 | 151.397 |
| 202.76 | 156.177 |
| 33.811 | 244.41 |
| 355.029 | 249.435 |
| 90.322 | 303.897 |
| 280.748 | 308.667 |
| 170.285 | 313.356 |
| 38.075 | 323.225 |
| 47.003 | 327.984 |
| 113.511 | 332.924 |
| 184.639 | 348.129 |
| 269.332 | 372.013 |
| 394.919 (least ranked surprising) | 376.529 |


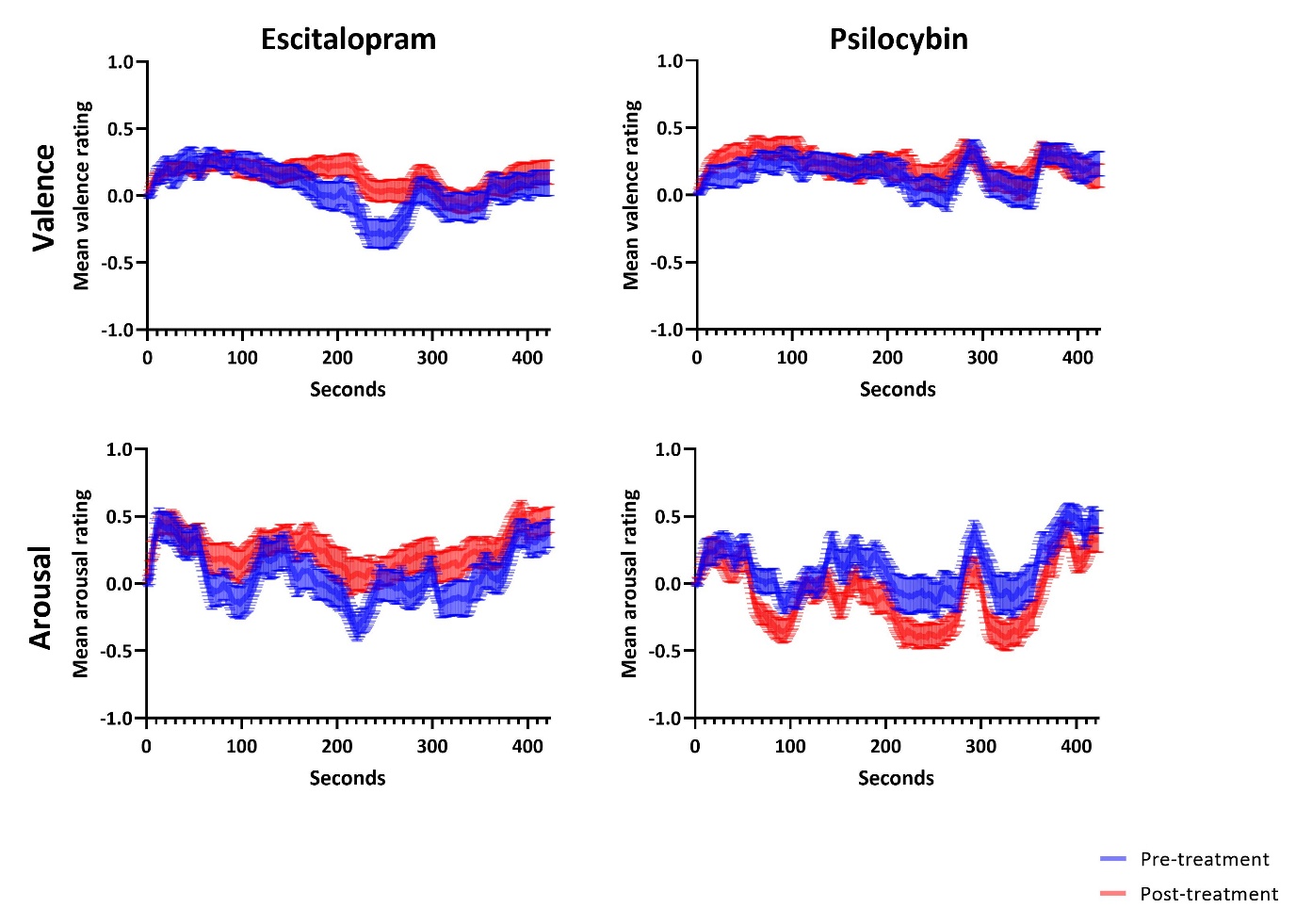
**
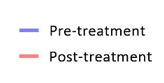
Figure S2. Average timecourses for valence and arousal**

The continuous mean ratings on the scales of valence (top panel) and arousal (bottom panel) are denoted for escitalopram (left panel) and psilocybin (right panel). Thickness of shading represents 1 SEM.

**Figure S3. Association of musical surprises with changes in arousal**


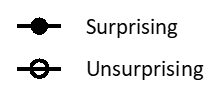
Results of a two-tailed paired t-test demonstrate that surprising events caused a significant increase in arousal compared to unsurprising events at pre-treatment in PT (t(21)=2.540; p=0.0191 ) although no difference in surprise-related arousal increase was seen post-treatment (p=0.2454) (Fig S3). Moreover, there was a notable surprise-related decrease in arousal in PT at both pre-treatment (t(21)=3.299; p=0.0034) and post-treatment (t(21)=3.515; p=0.0021). No significant change in affective response to surprising events was seen in escitalopram at pre- or post-treatment (p>0.05)

**Figure S4. Mean activation for each treatment group before and after intervention**

**ESC**

**PSI**


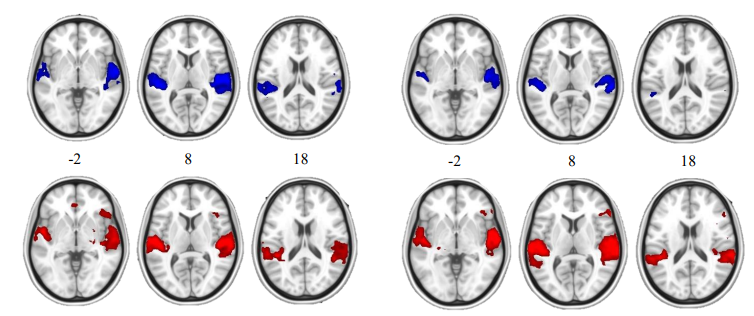

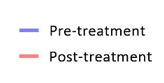


**Pre-treatment**

**Post-treatment**

Mean BOLD response to surprising>unsurprising events in ESC (n=19) (left panel) and PSI (n=22) (right panel) between pre (top panel; blue) and post-treatment (bottom panel; red). Results of voxelwise (Z> 2.3, p < 0.05, corrected for multiple comparisons) analysis shown in MNI space. As expected, cortical regions associated with music listening and auditory processing were activated during the task in all groups, including the superior temporal gyrus, Heschl’s gyrus and the planum temporale.

**Table S2. Characteristics of each treatment group**

|  | **Escitalopram** | | **Psilocybin** | |
| --- | --- | --- | --- | --- |
| **N (fMRI cohort)** | **19** | | **22** | |
| **Gender**  Male  Female | 13 6 | | 14 8 | |
| **Age (range) - yr** | 37.8 ± 10.3 (22-66) | | 41.7 ± 11.4 (21-64) | |
| **Endpoint** | **Pre-treatment** | **Post-treatment** | **Pre-treatment** | **Post-treatment** |
| **“How pleasurable did you find the music?” *Mean (SEM)*** | 8.105 (1.142) | 7.316 (0.9210) | 8.318 (1.003) | 9.045 (0.7399) |
| **GEMS subscales *Mean(SEM)***  Sublimity  Vitality  Unease | 2.446 (0.1083) 2.246 (0.1781) 1.803 (0.1560) | 2.358 (0.1441) 1.675 (0.1537) 1.658 (0.1636) | 2.542 (0.1608) 2.106 (0.1997) 1.750 (0.1854) | 2.682 (0.1209) 2.439 (0.1732) 1.864 (0.1715) |

Demographic characteristics of both treatment groups, including subjective ratings of the emotional experience (how pleasurable they found the music (measured on a visual analogue scale), subscales of the Geneva Emotional Musical Scales (GEMS) questionnaire). Details are provided for the fMRI cohort.

## Supplementary methods

### Continuous music ratings

Immediately after the fMRI session, participants were presented with the same piece of music and asked to continuously rate their emotional experiences using the EMuJoy software (1). Participants were asked to move a cursor with a computer mouse to indicate their real-time subjective feeling on the two-dimensional space that encompasses valence (i.e., pleasantness) on the horizontal axis, and arousal (i.e., activation) on the vertical axis, where activation was described as synonymous with arousal, in keeping with the classic two-dimensional model of emotion (2). Each cursor movement was recorded at a maximal rate of 20Hz.

**Musical-emotion label ratings** Immediately following the continuous ratings, participants were asked to complete the 25-item Geneva Emotional Music Scale (GEMS-25) (3)This questionnaire includes items that reflect musically induced emotional states. Participants indicated how strongly they experienced each of the items from 1-5 on a Likert Scale. Items are averaged into nine sub-factors of emotions: wonder, transcendence, power, tenderness, nostalgia, peacefulness, joyful activation, sadness and tension. These sub-factors contribute to 3 super-factors – vitality (power, joyful activation), unease (tension, sadness) and sublimity (wonder, transcendence, tenderness, nostalgia, peacefulness). This measure was taken to determine the general affective tone that was evoked in listeners.

### Subjective measures analysis

All statistical analysis was performed using Graphpad Prism version 9 (GraphPad Software, California, USA). A mixed-effects model was carried out to determine the interaction between *treatment* and *time* for each of the self-reported subjective measures, with subjects being modelled as random effects, and treatment and time as fixed. A *post hoc* paired (within-group) and unpaired (between-group) two-tailed t-tests were performed where a significant effect was observed.

**fMRI data pre-processing –** Four different but complementary imaging software packages were used to analyse the fMRI data. Specifically, FMRIB Software Library (FSL) (5), AFNI (6), Freesurfer (7) and Advanced Normalization Tools (ANTS) (8) were used. The preprocessing pipeline was previously validated by Carhart-Harris et al. (9) and was adapted based on Shany et al. (10). The stages are: 1) Removal of the first 24 volumes (30 sec) (FSLROI, FSL); 2) de-spiking (3dDespike, AFNI); 3) motion correction (3dVolreg, AFNI) by registering each volume to the first volume in the series; 4) brain extraction (BET, FSL); 5) rigid-body registration of functional image to structural image (FLIRT – Boundary Based Registration, FSL); 6) non-linear registration to 2mm Montreal Neurological Institute (MNI) standard brain (Symmetric Normalization, ANTs); 7) motion scrubbing-censoring (FSL Motion Outliers, FSL) – using a framewise displacement (FD) threshold = 0.4mm. The mean (±SD) FD was escitalopram: pre-treatment=0.113±0.048, post-treatment=0.107±0.032; PT: pre-treatment=0.133±0.06, post-treatment=0.006±0.026. The mean (±SD) percentage of volumes scrubbed was escitalopram: pre-treatment=0.67± 0.93%, post-treatment=0.11±0.19%; psilocybin therapy pre-treatment=0.68±2.58%, post-treatment=0.26±0.44% while the maximum percentage of censored volumes in one scan is 12.2%); 8) spatial smoothing (FWHM) using 6mm kernel (3dBlurInMask, AFNI); 9) high pass filter of 0.005 Hz (3dFourier, AFNI); 10) linear detrending (3dDetrend, AFNI); 11) regressing out 9 nuisance regressors (which underwent the same filter and detrending as in steps 9 and 10): 6 were motion-related (3 translations, 3 rotations) and 2 were anatomically-related (not smoothed). The anatomical regressors are 1) ventricles (Freesurfer, eroded in 2mm space), 2) draining veins (DV) (FSL’s CSF minus Freesurfer’s Ventricles, eroded in 1mm space) and 3) local white matter (WM) (FSL’s WM minus Freesurfer’s subcortical grey matter (GM) structures, eroded in 2mm space). Regarding local WM regression, AFNI’s 3dLocalstat was used to calculate the mean local WM time-series for each voxel, using a 25mm radius sphere centred on each voxel (11). ANTs symmetric non-linear normalization tool (SyN) was used in place of FSL’s non-linear registration tool FNIRT for registering functional images to MNI standard space (MNI152) due to higher accuracy across subjects and datasets (12).

**Mean task effect method –** An average activation was calculated for all participants from both pre and post treatment for responses to surprising versus unsurprising events. This was to determine the average effect of the task and to validate that the task activated regions that we would have expected. This was performed by averaging all first-level analyses files in FSL.

**Supplementary references**

1. Nagel F, Kopiez R, Grewe O, Altenmüller E. EMuJoy: Software for continuous measurement of perceived emotions in music. Behav Res Methods. 2007 May 1;39(2):283–90.

2. Thayer RE. Toward a psychological theory of multidimensional activation (arousal). Motiv Emot. 1978 Mar 1;2(1):1–34.

3. Zentner M, Grandjean D, Scherer KR. Emotions evoked by the sound of music: Characterization, classification, and measurement. Emotion. 2008;8:494–521.

4. Snaith RP, Hamilton M, Morley S, Humayan A, Hargreaves D, Trigwell P. A Scale for the Assessment of Hedonic Tone the Snaith–Hamilton Pleasure Scale. Br J Psychiatry. 1995 Jul;167(1):99–103.

5. Smith SM, Jenkinson M, Woolrich MW, Beckmann CF, Behrens TEJ, Johansen-Berg H, et al. Advances in functional and structural MR image analysis and implementation as FSL. NeuroImage. 2004 Jan 1;23:S208–19.

6. Cox RW. AFNI: Software for Analysis and Visualization of Functional Magnetic Resonance Neuroimages. Comput Biomed Res. 1996 Jun 1;29(3):162–73.

7. Fischl B. FreeSurfer. NeuroImage. 2012 Aug 15;62(2):774–81.

8. Avants BB, Tustison N, Johnson H. Advanced Normalization Tools (ANTS).

9. Carhart-Harris RL, Muthukumaraswamy S, Roseman L, Kaelen M, Droog W, Murphy K, et al. Neural correlates of the LSD experience revealed by multimodal neuroimaging. Proc Natl Acad Sci U S A. 2016 Apr 26;113(17):4853–8.

10. Shany O, Singer N, Gold BP, Jacoby N, Tarrasch R, Hendler T, et al. Surprise-related activation in the nucleus accumbens interacts with music-induced pleasantness. Soc Cogn Affect Neurosci. 2019 May 17;14(4):459–70.

11. Jo HJ, Saad ZS, Simmons WK, Milbury LA, Cox RW. Mapping sources of correlation in resting state FMRI, with artifact detection and removal. NeuroImage. 2010 Aug 15;52(2):571–82.

12. Klein A, Andersson J, Ardekani BA, Ashburner J, Avants B, Chiang MC, et al. Evaluation of 14 nonlinear deformation algorithms applied to human brain MRI registration. NeuroImage. 2009 Jul 1;46(3):786–802.
